# A Piezo-Electromagnetic Coupling Multi-Directional Vibration Energy Harvester Based on Frequency Up-Conversion Technique

**DOI:** 10.3390/mi11010080

**Published:** 2020-01-11

**Authors:** Ge Shi, Junfu Chen, Yansheng Peng, Mang Shi, Huakang Xia, Xiudeng Wang, Yidie Ye, Yinshui Xia

**Affiliations:** 1 College of Mechanical and Electrical Engineering, China Jiliang University, Hangzhou 310018, China; P1801085203@cjlu.edu.cn (J.C.); P1801085240@cjlu.edu.cn (Y.P.); 12b5100080@cjlu.edu.cn (M.S.); 2 Faculty of Electrical Engineering and Computer Science, Ningbo University, Ningbo 315211, China; xiahuakang@nbu.edu.cn (H.X.); 1611082572@nbu.edu.cn (X.W.); yeyidie@nbu.edu.cn (Y.Y.); xiayinshui@nbu.edu.cn (Y.X.)

**Keywords:** piezo-electromagnetic coupling, up-conversion, vibration energy harvester, multi-directional vibration, low frequency vibration

## Abstract

Harvesting vibration energy to power wearable devices has become a hot research topic, while the output power and conversion efficiency of a vibration energy harvester with a single electromechanical conversion mechanism is low and the working frequency band and load range are narrow. In this paper, a new structure of piezoelectric electromagnetic coupling up-conversion multi-directional vibration energy harvester is proposed. Four piezoelectric electromagnetic coupling cantilever beams are installed on the axis of the base along the circumferential direction. Piezoelectric plates are set on the surface of each cantilever beam to harvest energy. The permanent magnet on the beam is placed on the free end of the cantilever beam as a mass block. Four coils for collecting energy are arranged on the base under the permanent magnets on the cantilever beams. A bearing is installed on the central shaft of the base and a rotating mass block is arranged on the outer ring of the bearing. Four permanent magnets are arranged on the rotating mass block and their positions correspond to the permanent magnets on the cantilever beams. The piezoelectric cantilever is induced to vibrate at its natural frequency by the interaction between the magnet on cantilever and the magnets on the rotating mass block. It can collect the nonlinear impact vibration energy of low-frequency motion to meet the energy harvesting of human motion.

## 1. Introduction

Intelligent wearable devices have become a new application hotspot with the rapid development of electronic technology [1]. However, it is difficult for the battery of intelligent wearable devices to meet the requirements of its constantly improving functions and long-time standby [2,3]. With the development of ultra-low power technology and micro-electro-mechanical system (MEMS) technology, it is possible to harvest environmental energy to supply power for intelligent wearable devices [4,5,6]. At present, the energy that can be used in the environment includes solar energy, heat energy, vibration energy and so forth, and vibration energy widely exists in the human living environment [7,8,9]. Therefore, researchers have begun to study the technology of harvesting vibration energy in the environment and converting it into electrical energy to power intelligent wearable devices.

At present, the most studied environmental vibration energy harvesting methods are the piezoelectric harvester and the electromagnetic harvester. The output voltage of the piezoelectric vibration energy harvester is higher but the output current is smaller and its output impedance is capacitive [10,11,12]. The output voltage of electromagnetic vibration energy harvester is lower but the output current is larger and its output impedance is inductive [13,14,15,16]. The output power and conversion efficiency of the energy harvester with a single electromechanical conversion mechanism is low and the operating frequency band and load range are narrow [17,18]. Because of the resonance type, a linear vibration energy harvester has higher output power only near its natural resonance frequency point [19]. The smaller the harvester size is, the higher the natural resonant frequency is. At present, the vibration in the range of human application is mostly low-frequency vibration [20,21]. To harvest low-frequency vibration energy, it is necessary to convert the low-frequency vibration to the natural resonant frequency vibration of harvester.

Researchers have also proposed many new structures for vibration energy harvesting. Li et al. [22] designed an enhanced bandwidth nonlinear resonant electromagnetic energy harvester. The inertial mass of the proposed harvester is formed by four stacked ring permanent magnets (PMs), which is suspended axially by two magnetic springs and circumferentially by ferrofluid within a carbon fiber tube. The magnetic springs are made up of two button PMs adhered, respectively, to the end cap at each end of the carbon fiber tube to provide varying repulsive forces to the PM stack, resulting in enhanced resonance frequency band and higher efficiency in energy harvesting. However, this kind of energy harvester can only collect vibration energy in a certain direction [15,22], which has no obvious advantage for human motion or swing. Therefore, many researchers use rotating mass structure to harvest human motion energy [21,23,24]. For example, Smilek et al. [23] present a novel design of a nonlinear kinetic energy harvester for very low excitation frequencies below 10 Hz. The design is based on a proof mass, rolling in a circular cavity in a Tusi couple configuration. This allows for an unconstrained displacement of the proof mass while maintaining the option of keeping the energy transduction element engaged during the whole cycle and thus reducing the required number of transduction elements. Romero et al. [21] present a micro-rotational energy harvester topology for extracting electric energy from human body motion at joint locations. This was accomplished using an inertial-based axial flux machine constructed with multiple permanent magnet poles and stacked micro-fabricated planar coils. An average power of 472 µW was obtained when the 2 cm^3^ device was placed on the ankle while walking at 4mph. Some researchers use an up-conversion method to convert external low-frequency excitation to high-frequency vibration of cantilever beam to harvest energy [19,25,26]. For example, Gu et al. [19] present an energy harvesting device in which a low frequency resonator impacts a high frequency energy harvesting resonator, resulting in energy harvesting predominantly at the system’s coupled vibration frequency. Pillatsch et al. [25] present an energy harvester specifically targeted at low frequency random excitation as is encountered in human motion. The design uses a frequency up-conversion technique based on magnetic actuation of a piezoelectric beam. The benefits of a rotational design with eccentric proof mass compared to a linear design are discussed. This makes the approach suitable for the random device orientations of the human body. Researchers also try to achieve efficient energy harvesting through the combination of various energy exchange mechanisms [27,28,29,30]. For example, Zhu et al. [27] present the design and analysis of a new magnetoelectric energy harvester that uses Terfenol-D/piezoelectric/Terfenol-D laminate to harvest energy from nonlinear vibrations created by magnetic levitation. Due to the high energy density and strong magneto-mechanical coupling effect of magnetostrictive material, the proposed harvester is capable of generating very high voltage and power at low frequency ranges. Ibrahim et al. [29] proposed two hybrid energy harvesters that each employs a combination of piezoelectric (PZT), magnetostrictive (MSM) and electromagnetic technologies. Harvesters employ spiral geometries to allow smaller natural frequencies compared to a straight beam. In particular, the hybrid technology that uses piezoelectric and magnetostrictive has shown significant improvement in the frequency bandwidth of the device.

As mentioned above, most of the methods of human motion energy harvesting adopt a rotating mass structure or up-conversion technology. Up-conversion technology can be divided into collision contact type [19] and non-contact type [25,26]. The non-contact type mainly uses the magnetic force to excite the piezoelectric cantilever beam to generate resonance. However, they also have some defects, for example, they cannot harvest vibration energy in multiple directions; the magnetic method limits the swing amplitude of the cantilever; the driving force of piezoelectric plate vibration only has one direction; the voltage of the electromagnetic coil is too low, which will bring high requirements to the subsequent energy harvesting circuit.

In this paper, a new energy harvester that combines piezoelectric electromagnetic coupling resonance and up-conversion technology is proposed. By composing a multi field nonlinear coupling system of force-electricity-magnetism, a higher working frequency band can be obtained. The up-conversion technology is used to harvest low frequency vibration energy and rotating mass block is used to drive piezoelectric cantilever beam to obtain multi-directional energy harvesting. This paper mainly discusses the piezoelectric electromagnetic coupling model, analyzes the factors that affect the power of the system and tests its energy collection effect on the experimental device.

## 2. Design of Energy Harvester

**Figure 1:**
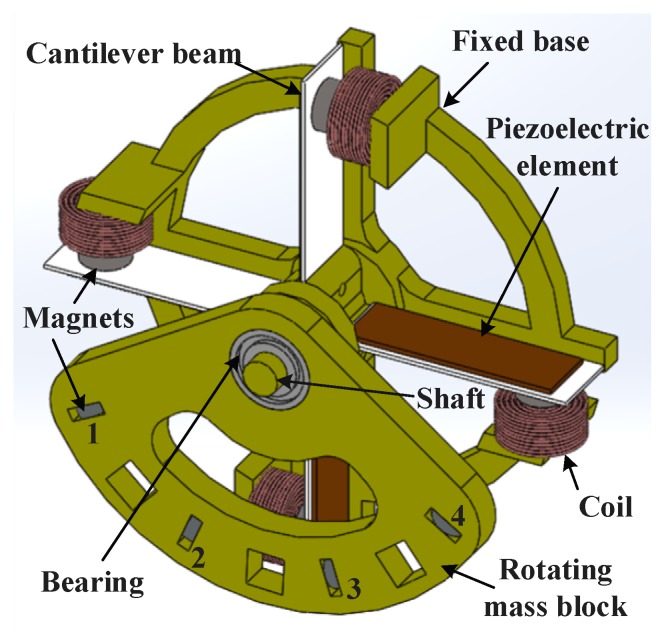
The structural design of the multi-directional vibration energy harvester.

It is common to harvest the low-frequency vibration energy of the human body through low-frequency rotating motion. In this paper, a new structure of piezoelectric electromagnetic coupling multi-directional vibration energy harvester based on a frequency up-conversion technique is proposed. The structural design of the energy harvester is shown in Figure 1.

Four piezoelectric electromagnetic coupling cantilever beams were installed on the axis of the base along the circumferential direction. Piezoelectric elements were set on the surface of each cantilever beam to harvest piezoelectric energy. The permanent magnet on the beam was placed on the free end of the cantilever beam as a mass block. Four coils for collecting energy were arranged on the base under the permanent magnets on the cantilever beams to harvest electromagnetic energy. A bearing was installed on the central shaft of the base and a rotating mass block was arranged on the outer ring of the bearing. Four permanent magnets were arranged on the rotating mass block and their positions corresponded to the permanent magnets on the cantilever beams.

The permanent magnets used in the harvester were Neodymium Iron Boron magnets (NdFeB) permanent magnets (Φ6 mm × 3 mm) and were arranged so that they were mutually exclusive as this proved to be advantageous. The bearing model was PNY-686ZZ (SAKAGAMI, Tokyo, Japan), with an inner diameter of 6 mm, an outer diameter of 13 mm and a thickness of 5 mm. It was mounted on the 6 mm central axis of the base. The rotating mass block was made of polylactic acid (PLA) material by way of 3D printing. The diameter of the circular hole matched with the outer ring of the bearing was 6mm, the thickness is 5 mm too and the weight is 30 g. Mass block can be added on it to increase its inertia. The base is also made of PLA material by way of 3D printing. The fixed base was in the shape of a ring and an outer ring was connected by four pillars, with a diameter of 70 mm. It provided four platforms for coils. The macro-fiber composite (MFC) piezoelectric elements (M-2807-P2, Smart Material, Sarasota, FL, USA) were glued to the cantilever beams. The capacitance of the piezoelectric was 14.3 nf and the tested natural frequency of the cantilever beam was about 42 Hz. The small scale device can be realized by reducing the size of the cantilever beam, the area of the piezoelectric, the volume of the coil and so forth.

## 3. Principle and Model Analysis

**Figure 2:**
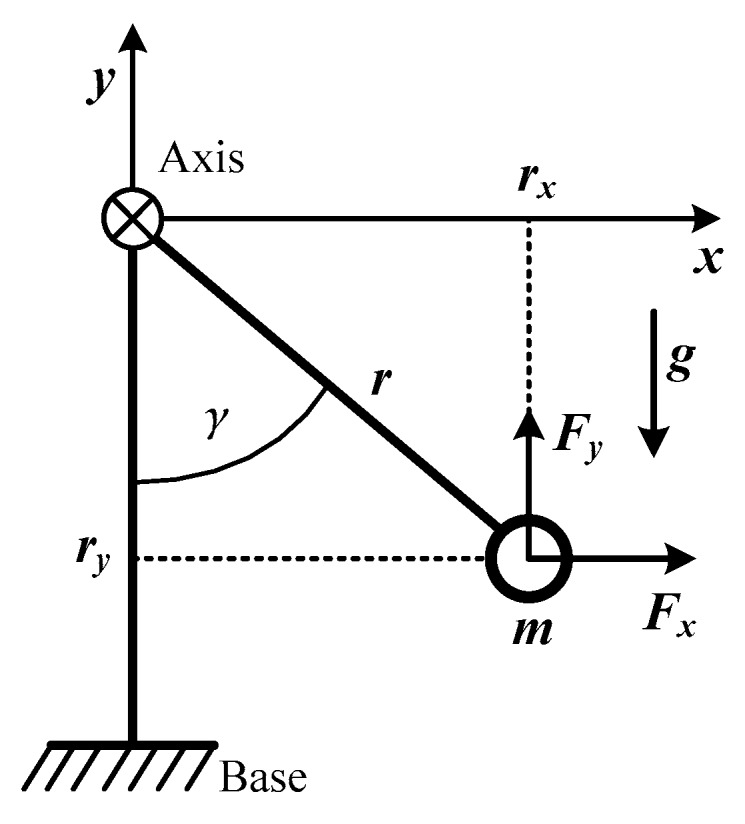
Schematic view of the rotating mass under external excitation.

In order to further understand the principle and dynamic behaviour of this harvester, the configuration depicted in Figure 2 was studied. A point mass m at a distance *r* from its axis of rotation was considered under gravity and external excitation. The motion equation of the rotating mass was as shown in Equation (1) [25].

(1) $$\ddot{\gamma }I=F_{x}r_{y}+(F_{y}-mg)r_{x},$$

where the distances *r_x_* and *r_y_* describe the rotating mass position and *F_x_* and *F_y_* are the corresponding inertial reaction forces caused by linear external excitation. *I* = *mr*^2^/4 is the mass moment of inertia of the rotor. Gravity g acts in the negative y-direction. The angle *γ* is the angular deflection of the proof mass in relation to the y-axis.

Through Equation (1), it can be clearly seen that the rotation design can accept the linear and rotation excitation in *x* and *y* directions, which makes it more versatile in the case of variable equipment direction and host motion in multiple degrees of freedom at random, just like in the human body. Under the excitation of gravity and rotation, the rotating mass behaves like a pendulum. If the base is rotated, the rotating mass only stays in the negative y-direction, resulting in *γ*′ = −*γ*. The relative motion between the base and the rotor becomes *γ*′(*t*) = −*γ*(*t*). In this scenario the device is behaving purely inertia. In most real applications there will be a mixture of these cases of pendulum and inertial motion. However, the basic analysis method is the same.

**Figure 3:**
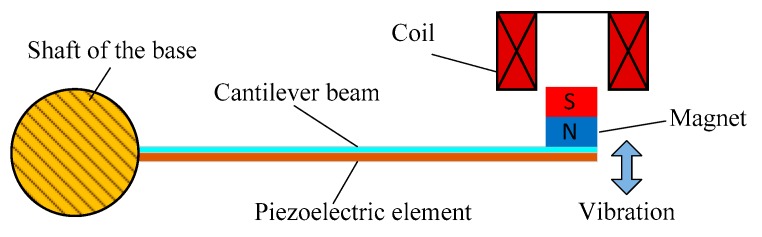
Structural diagram of single piezoelectric electromagnetic coupling cantilever beam.

The structure diagram of a single piezoelectric electromagnetic coupling cantilever beam is shown in Figure 3. The surface of the cantilever beam was pasted with MFC piezoelectric element and the free end of the cantilever beam is provided with permanent magnet and coil. When the cantilever beam vibrates under external excitation, the MFC piezoelectric element will output alternating voltage because of alternating stress and the closed coil will also produce alternating current because of the change of magnetic flux. The former is based on the positive piezoelectric effect and the latter is based on Faraday’s electromagnetic induction law. *U* is the vibration displacement of cantilever beam. The piezoelectric electromagnetic coupling vibration energy harvester proposed in this paper improves the harvesting efficiency by integrating the two electromechanical conversion mechanisms to a cantilever beam structure.

**Figure 4:**
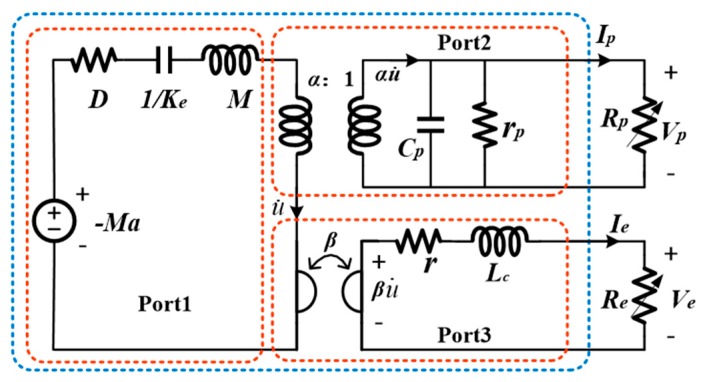
Equivalent circuit model of a single piezoelectric electromagnetic coupling cantilever beam. Port1: mechanical domain, Port2: piezoelectric unit, Port3: electromagnetic unit.

The equivalent circuit model of a single piezoelectric electromagnetic coupling cantilever beam is shown in Figure 4.

Here, *Ma* represents the external exciting force in the mechanical domain and the resistance represents the damping *D*, the capacitance represents the elastic potential energy, which is expressed by 1/*K_e_* of the elastic coefficient, the inductance represents the kinetic energy, which is expressed by the magnet mass *M*. The current of the primary circuit is the vibration velocity u of the mass relative to the base. The equivalent circuit of piezoelectric element is coupled to mechanical domain through transformer, while the equivalent circuit of electromagnetic element is coupled to the mechanical domain through a gyrator [18]. The *α* represents the piezoelectric force-voltage factor and *β* the electromagnetic force-current factor. In the piezoelectric equivalent circuit, the *C_p_* and *r_p_* are corresponding clamped capacitor and internal resistance of the piezoelectric element. Because of the large value of *r_p_*, it is often ignored in analysis. In the electromagnetic element circuit, the *L_c_* and *r* are the corresponding inductance and internal resistance of the electromagnetic coil.

When the output ports of the piezoelectric element and electromagnetic coil are respectively connected to two resistance loads *R_p_* and *R_e_*, *V_p_* and *V_e_* are the corresponding output voltages of the piezoelectric element and electromagnetic coil; *I_p_* and *I_e_* are corresponding to the output currents of the piezoelectric element and electromagnetic coil.

Assuming that the excitation acceleration is single sine wave *a*(*t*) = *a*_M_sin(*ωt*) (0 ≤ *t* ≤ 2π/*ω*) for simple analysis, the *a*_M_ is the amplitude of acceleration, the *ω* is angular frequency of rotating mass block. The relative movement displacement between the free end of the cantilever and the base is *u*(*t*), the system differential equation can be described as [31]:

(2) $$M\ddot{u}(t)+D\dot{u}(t)+K_{e}u(t)=-Ma(t)-\alpha V_{p}(t)-\beta I_{e}(t),$$

According to Kirchhoff’s law, the electrical equations are established as follows:

(3) $$\alpha \dot{u}(t)=C_{p}{\dot{V}}_{p}(t)+\frac{V_{p}(t)}{R_{p}},$$

(4) $$\beta \dot{u}(t)=L_{e}{\dot{I}}_{e}(t)+(R_{e}+r)I_{e}(t).$$

The transfer function relationship between the relative movement displacement *U*(*s*) of the free end of the cantilever beam and the excitation acceleration *A*(*s*) of the foundation can be obtained by the Laplace transformation and simplification under the zero initial condition of Equation (1), Equation (2) and Equation (3):

(5) $$\frac{U(s)}{A(s)}=\frac{-M}{Ms^{2}+Ds+K_{e}+\beta^{2}s/(r+R_{e}+L_{c}s)+\alpha^{2}R_{p}s/(1+C_{p}R_{p}s)}.$$

By calculating the amplitude of the transfer function, the maximum amplitude of the free end of the cantilever at vibration frequency *ω* can be obtained:

(6) $$u_{M}=\frac{Ma_{M}}{\sqrt{{\left[-M\omega^{2}+K_{e}+\frac{\omega^{2}\beta^{2}L_{c}}{{\left(r+R_{e}\right)}^{2}+\omega^{2}L_{c}{}^{2}}+\frac{\omega^{2}R_{p}{}^{2}\alpha^{2}C_{p}}{1+{\left(\omega C_{p}R_{p}\right)}^{2}}\right]}^{2}+{\left[D\omega +\frac{\beta^{2}(r+R_{e})\omega }{{\left(r+R_{e}\right)}^{2}+\omega^{2}L_{c}{}^{2}}+\frac{\alpha^{2}R_{p}\omega }{1+{\left(\omega C_{p}R_{p}\right)}^{2}}\right]}^{2}}}.$$

Therefore, each time the magnet of the rotating mass block and the magnet on the cantilever beam pass by, the cantilever will be excited to its maximum amplitude *u_M_* position and then the cantilever will attenuate the vibration with its own natural frequency.

It can be seen from Equation (6) that the electromagnetic element mainly affects the damping of the harvester, while the piezoelectric element also affects the stiffness and damping of the harvester. The output power of the piezoelectric energy harvesting unit in the piezoelectric electromagnetic coupling energy capture device [18,31] is not only affected by the structural parameters and piezoelectric material properties but also by the characteristic parameters of the electromagnetic energy harvesting unit. Similarly, the output power of the electromagnetic energy capture unit is also affected by the characteristic parameters of the piezoelectric unit. Therefore, the appropriate transducer structure size and interface circuits will effectively improve the conversion efficiency of vibration energy harvester.

There are two benefits to using the converters combination. First, since permanent magnets are used in the up-conversion technology of piezoelectric cantilevers, an additional electromagnetic energy harvesting method is added to the existing permanent magnets, which can improve the conversion efficiency of vibration energy. In addition, the optimal load range (set 0.9 peak power band) of a single conversion mechanism is smaller. In the combination of two converters, the corresponding piezoelectric and electromagnetic loads can have more load combinations to obtain the same total power (also 0.9 peak power band) [18]. Therefore, it is easier to achieve maximum power tracking.

## 4. Experimental Test

In order to test the performance of the vibration energy harvester designed in this paper, two motion platforms were designed to test the experimental prototype.

**Figure 5:**
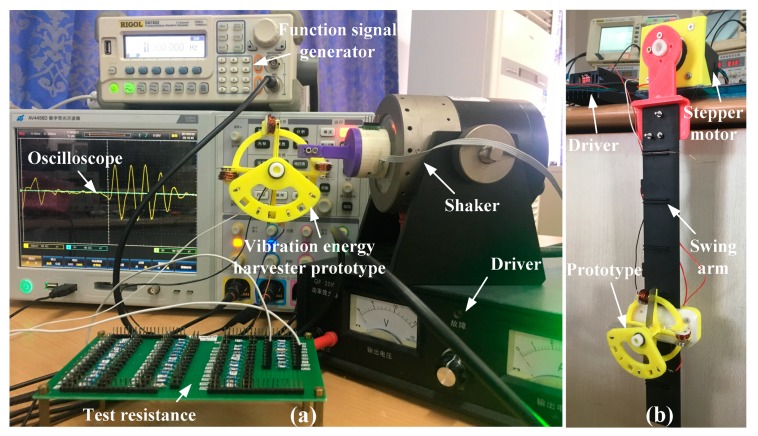
Experimental setup. (**a**) Single dimension vibration test platform. (**b**) Swing device for simulating the swing of human arm.

The first was a single dimension vibration test platform, as shown in Figure 5a, which was composed of a shaker (ZJ-2A, Shanghai Zhurui CO., Shanghai, China), a driver (GF-20W, Shanghai Zhurui CO., Shanghai, China) and a function signal generator (DG3121A, RIGOL CO., Beijing, China). It can simulate a single dimension excitation vibration. Different vibration conditions can be simulated by controlling its vibration amplitude and frequency. By changing different frequency and amplitude, the motion state of the energy harvester under different excitation conditions was obtained and the results under different experimental conditions were obtained.

The second test platform was a swing device for simulating the swing of human arm, as shown in Figure 5b. It is a swing device composed of a stepper motor and a driver, which can simulate the low frequency and large swing characteristics of human arm. It can simulate different motion conditions by controlling its swing amplitude and speed. By changing different acceleration, frequency and swing amplitude, the motion state of the energy harvester under different motion conditions can be obtained and the results under different experimental conditions can be obtained.

The excitation source used in the test was a sinusoidal acceleration signal, the acceleration amplitude was 0.8 g and the excitation frequency was 4.8 Hz. The resistance range used in the test was 1.3 Ω–3.2 MΩ, in which the maximum power output matching resistance of piezoelectric was about 100 KΩ and the maximum power output resistance of coil is 80 Ω.

### 4.1. Excitation Test of Permanent Magnets

There are four positions (1, 2, 3, 4) on the rotating mass block of the harvester that can be installed with permanent magnets, as shown in Figure 1. The harvester with different number of permanent magnets was tested. The voltage of piezoelectric element just reflects the vibration of cantilever, so the voltage of piezoelectric element was tested.

**Figure 6:**
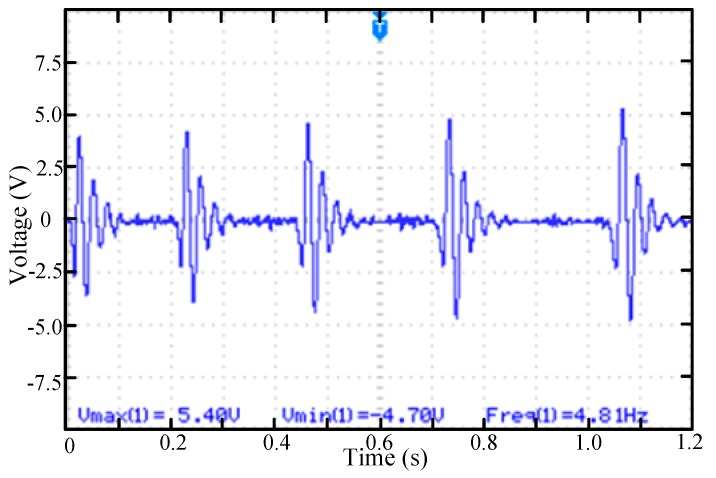
The voltage waveform of the piezoelectric beam while rotating mass block rotates with a permanent magnet (position 1).

The test platform used was the swing device for simulating the swing of human arm as shown in Figure 5b. The frequency of swing was 4.8 (Hz). The first case test was to install only one permanent magnet. The permanent magnet was installed at position 1. The maximum output voltage amplitude of a single piezoelectric element can reach 5.4 V. As shown in Figure 6, the cantilever beam moves to the maximum amplitude after being repulsed by the permanent magnet on the rotating mass block. After the permanent magnet on the rotating mass and the permanent magnet on the cantilever beam passing by, the cantilever beam begins to vibrate at the natural frequency of the cantilever beam about 42 (Hz), and the amplitude of the vibration decreases continuously. Every time of the two permanent magnets passing by will cause up-conversion vibration of the cantilever. The low frequency vibration was converted to the inherent high frequency vibration of the cantilever beam.

**Figure 7:**
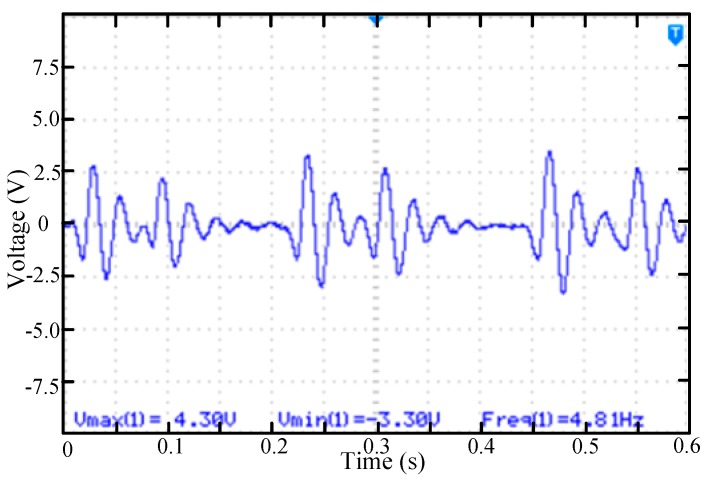
The voltage waveform of the piezoelectric beam while rotating mass block rotates with two permanent magnets (positions 1, 4).

In the second case, permanent magnets were installed at positions 1 and 4 of the rotating mass block. Other test conditions remained unchanged from the first test. As shown in Figure 7, there will be two up-conversion processes each time the rotating mass passes passing by the cantilever beam. Because the rotating mass has a fast swing speed, the vibration excited by the first permanent magnet has not been attenuated and the excitation of the second permanent magnet will be added again. The natural vibration frequency of the cantilever is constant and the vibration duration is nearly twice as long.

**Figure 8:**
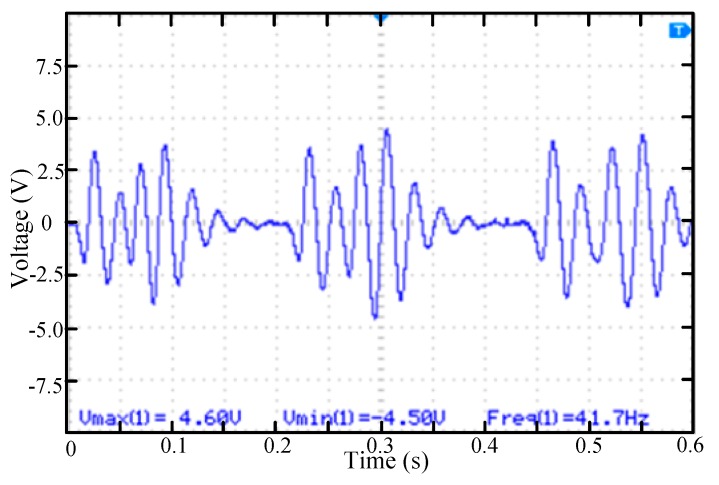
The voltage waveform of the piezoelectric beam while rotating mass block rotates with three permanent magnets (positions 1, 3, 4).

In the third case, the permanent magnet was set at the positions 1, 3 and 4 of the rotating mass block. As shown in Figure 8, since the permanent magnet at position 3 was increased compared with the second case and the distance between 1 and 3 was relatively large, the vibration amplitude was attenuated after the position 1 magnet passed by. However, the distance between the magnets at positions 3 and 4 was relatively close. It can be found that the excitation of the magnet at the position 3 has not yet decayed and the excitation of the position 4 magnet is superimposed to positions 3. So in the wave form, the vibration amplitude of cantilever beam increases again from position 3 and then starts to decay after the position 4 magnet passing by.

**Figure 9:**
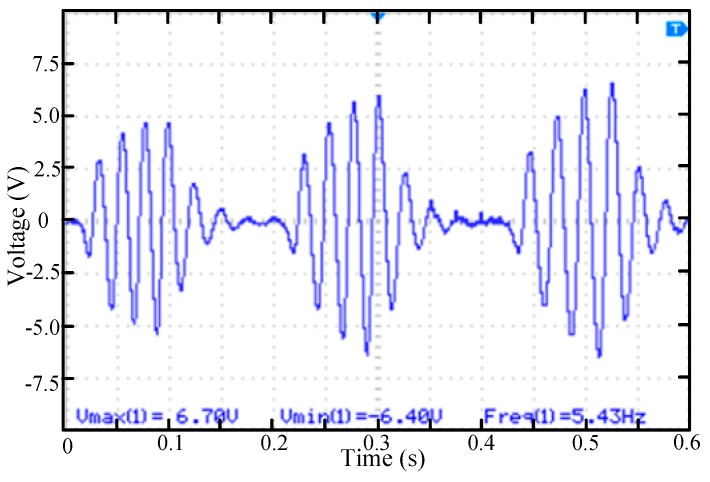
The voltage waveform of the piezoelectric beam while rotating mass block rotates with four permanent magnets (positions 1, 2, 3, 4).

In the fourth case, permanent magnets were installed at all four positions. As shown in Figure 9, the continuous excitation of the four permanent magnets made the cantilever begin to vibrate under the excitation of the first permanent magnet and the vibration amplitude increased continuously. The maximum output amplitude of piezoelectric element can reach 6.7 V and the whole up-conversion vibration process also lasts longer. Therefore, in this case, the harvester can harvest more vibration energy than in the first case.

### 4.2. Single Direction Weak Vibration Test

**Figure 10:**
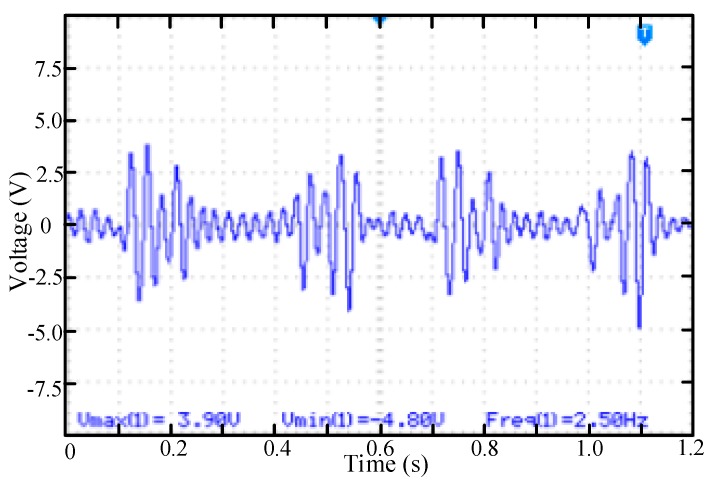
The voltage waveform of the piezoelectric beam while rotating mass block low amplitude reciprocating swing.

In many cases, the vibration is weak and the direction of vibration is single. The test device is the single dimension vibration test platform as shown in Figure 5a. Under this excitation condition, only the permanent magnets at positions 2 and 3 can repeatedly excite the piezoelectric cantilever beam. The vibration frequency is 2.5 Hz. The measured voltage waveform is shown in Figure 10. In this case, the cantilever has been in the state of repeated excitation and there is a large amplitude vibration when the permanent magnet passing by and there is a low amplitude vibration when there is no permanent magnet passing by.

In this case, the design of interface circuit is more critical. By harvesting the energy of weak vibration, the conversion efficiency of the whole vibration energy can be improved.

### 4.3. Piezo-Electromagnetic Coupling Output Test

**Figure 11:**
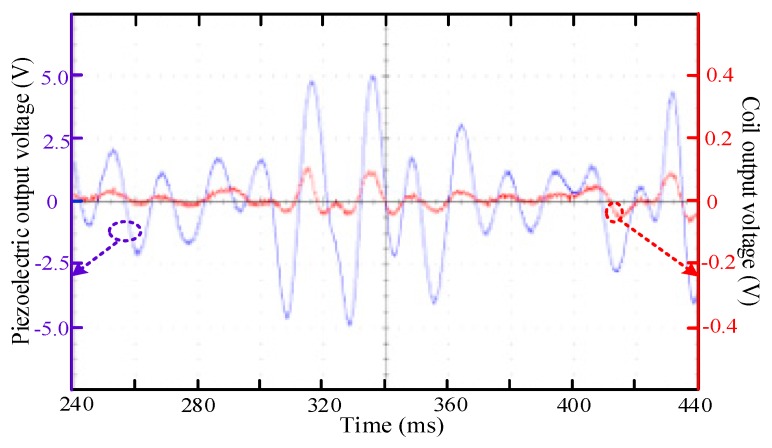
The output voltage wave forms of the piezoelectric beam and coil while rotating mass block random swing.

The harvester adopts the cantilever beam of the piezoelectric and electromagnetic coupling mode. In the process of cantilever vibration, the vibration of the permanent magnet at the end of the cantilever beam will change the magnetic flux of the coil under the permanent magnet, so that the coil can output alternating current. The diameter of the enameled wire used in the coil was 0.1 mm and the number of turns was 200. Due to the limitation of volume, if the thinner enameled wire is used, the internal resistance will be increased and the internal power consumption of the coil will be increased. With a thicker enameled wire, the number of turns of the coil will be fewer and the corresponding output voltage will be lower. According to Faraday electromagnetic induction, the voltage is related to the change rate of magnetic flux. The greater the speed of vibration, the greater the output voltage of the coil. However, the size of cantilever determines the maximum vibration amplitude. As shown in Figure 11, the output voltage of the coil is low and its voltage amplitude is about 100 mV. The frequency of the output voltage of the solenoid is the same as that of the piezoelectric because the coil and piezoelectric cantilever are coupled and resonant. The output frequency of the coil is determined by the vibration frequency of the cantilever. Because of the coupling resonance, the trend of output voltage waveform of the coil is consistent with that of the piezoelectric output. With the up-conversion of the cantilever beam, it first increases and then decreases.

The magnetic field produced by the coil current will react on the permanent magnet of cantilever beam and affect the vibration amplitude of the cantilever beam. It can be seen from Figure 11 that the vibration of the piezoelectric cantilever beam is less affected by the smaller output voltage amplitude of the coil. However, the energy output of the coil is increased. Therefore, the overall energy collection efficiency will be improved by using an efficient low-voltage energy harvesting circuit or using the output energy of the coil as the auxiliary energy for the piezoelectric cantilever energy harvesting.

The maximum power matching test was carried out with different resistors, as shown in Figure 5a. When the piezoelectric device acted alone, the peak power of the energy harvesting was 1.28 mW. When the electromagnetic energy acted alone, the peak power was about 0.03 mW. When piezoelectric and electromagnetic are coupled, the peak power was about 1.31 mW. The power of energy collection was the highest with a better energy conversion effect when there was piezoelectric and electromagnetic coupling resonance.

The permanent magnets and coils used in the prototype were small and the number of turns of as coils is relatively few, while the piezoelectric used in the prototype was relatively large, so the energy extracted by the two transducers was quite different. It can redistribute the harvested energy, such as 50%/50% or other proportions by reducing the size of the piezoelectric elements, thickening the coils to reduce the internal resistance, increasing the number of turns to increase the output voltage and using stronger permanent magnet.

## 5. Discussion

At present, there are various kinds of energy harvesters and some novel structures are selected for comparison and discussion. The main characteristics are shown in Table 1. In the structure of Reference [21], printed circuit board (PCB) coil is used to cut the magnetic field line of permanent magnet to generate electricity. It can harvest the energy of the human swing motion process but the energy it harvests is relatively low. In Reference [27], a new kind of vibration energy harvester which combines magnetostrictive material and piezoelectric material is used but it can only harvest single direction vibration and the power density provided in the paper only considers the volume of magnetoelectric laminate composites (MLCs). In Reference [11], the piezoelectric cantilever is excited by permanent magnet but it can only harvest the energy of rotation excitation and the harvesting energy power is low. Compared with the structures proposed in these papers, the harvester in the current paper used four permanent magnets to continuously excite the cantilever beams, which can produce continuous vibration with large amplitude. Four cantilever beams were used to harvest vibration energy in multiple directions. With the coupling of electromagnetic and piezoelectric, more vibration energy can be extracted when the appropriate interface circuit is selected.

## 6. Conclusions

In this paper, a new structure of piezoelectric electromagnetic coupling up-conversion multi-directional vibration energy harvester is proposed. Four piezoelectric electromagnetic coupling piezoelectric cantilever beams are installed on the axis of base along the circumferential direction. The permanent magnet on the beam is placed on the free end of the cantilever beam as a mass block. Four coils for collecting energy are arranged on the base under the permanent magnets on the cantilever beams. Four permanent magnets arranged on the rotating mass block continuously excite the cantilever beams. The piezoelectric cantilever is induced to vibrate at its natural frequency by the interaction between the magnet on cantilever and the magnets on the rotating mass block. With the coupling of electromagnetic and piezoelectric, more vibration energy can be extracted when the appropriate interface circuit is selected.

## Figures and Tables

**Table 1:** Comparison of several vibration energy collection structures.

Literature	GTD2019 [11]	TMAGN2012 [27]	MEMS2011 [21]	This Work
Vibration excitation mode	Permanent magnet excitation	Magnetostriction	Permanent magnet excitation	Permanent magnet excitation
Energy harvesting method	Piezoelectricity	Piezoelectricity	Electromagnetism	Piezo-electromagnetic coupling
Maximum Output power	234.47 μW	1.1 mW	472 μW	1.31 mW
Power density ^1^	Not available	3.46 × 10^3^ W/m^3^ (MLCs)	0.236 W/m^3^	11.35 W/m^3^
Operating frequency	0.167–1.677 Hz	10 Hz	<10 Hz	1–15 Hz
Directional of vibration	Rotational	Single direction	Swing	Multidirection vibration and swing
Frequency up-conversion	Yes	No	No	Yes

^1^ The result is based on the information provided.
